# Selection of suitable knitting parameters for 1 x 1 rib collar manufacturing in V-bed knitting machine

**DOI:** 10.1016/j.heliyon.2021.e06545

**Published:** 2021-03-23

**Authors:** Md. Shakhawat Hossain, MD. Momtaz Islam, Naimul Hasan

**Affiliations:** a Department of Textile Engineering, Khulna University of Engineering & Technology, Khulna, 9203, Bangladesh; b Department of Fabric Engineering, Bangladesh University of Textiles, Tejgaon, Dhaka, 1208, Bangladesh

**Keywords:** Equation, Cost minimization, Required size, Ply, Needle, Flat knitted collar

## Abstract

The main components of a polo shirt are the body and collar. For different body measurements, different collar sizes are needed. It is very crucial to select the perfect collar size according to body size; otherwise, collar size will be larger or smaller than the required size. However, producing and maintaining perfect collar size concerning body size is very tough as the collar is tiny. If the finished collar size is larger or smaller than the appropriate size, the manufacturer is supposed attach the incorrectly measured collar to the body, placing the customer at risk of a vital quality argument. Otherwise, the manufacturer would have to remake the collar, wasting both time and money. Sometimes, the knitting industry has to deal with purchase order cancellations due to a lack of lead time for replication. To avoid the complexities described earlier, quantitative equations for collar production based on the number of ply, stitch duration, and needle count were established in this study. The precision of this exploration work is approximately 100 percent for matching exact collar size with body size. As a result, the evolved technique can be used in the textile knitting industry to ensure that accurate specifications are met the first time.

## 1 Introduction

Knit fabric has some special properties like comfortable to wear, relatively easy to make and work with, beautiful texture, resistance to wrinkling and elasticity [1]. Knit fabric may be classified as Single jersey, Double Jersey and Special fabric. Single Jersey has some derivatives that are Single Lacoste, Double Lacoste, Single Pique, Polo pique or Double pique, cross miss, and many more [2]. The final product by these derivatives may be a Polo shirt, T-Shirt, etc [3]. Body, collar and cuff are the main components of polo shirt. Single Lacoste, double Lacoste, single pique, Polo pique or Double pique are mainly used for body fabric. On the other hand, 1 × 1 rib is mainly used for collar & cuff which is produced by v-bed flat knitting machine [2]. Polo shirt having different sizes and measurements such as S (Small) size, M (Medium) size, L (Large) size, XL size, etc. For this reason, collar and cuff size also varies according to body size [4]. Body fabric (Single lacoste, double Lacoste, single pique, Polo pique or double pique) is produced in single jersey circular knitting machine and collar is produced in V-bed flat knitting machine [5]. Normally, 180–240 GSM ranges fabric are used as body fabric of polo shirt and required GSM of collar is 1.5–2.5 times of the body fabric. It should be mentioned that the yarn count of body fabric and collar fabric should be the same because a similar yarn count facilitates shade matching between body and collar fabric after the dyeing as well as provide equal elasticity and hand feel [6, 7, 8, 9].

Besides, it is exceptionally hard to keep up exact collar size as per body size in the wake of completing as a result of its shrinkage properties because of various number of number of ply, yarn count and stitch length. The completed collar size is expanded by 0.5 cm–0.8 cm after the expulsion of the separation thread. Furthermore, the collar size is expanded by 0.5 cm after the connection with the body parts by the sewing activity. The applied pressing factor by the sewing machine and the pulling out by the sewing operator during the sewing, permit the collar to be stretched up to 0.5 cm. Consequently, completed size of the collar ought to be around 1.0 cm less than the required size. In this way, it very well may be said that, to get exact required size, completed size ought to be fixed and that relies upon certain boundaries, for example, stitch length, number of ply and quantities of needles utilized [10, 11, 12, 13, 14, 15, 16].

The current practice in the apparel business is to follow the grading process whenever completed size is bigger or smaller than the exactly needed size. The grading process implies that if the completed collar doesn't coordinate with the necessary size, attempting to coordinate with the bigger or smaller one. The main goal of this research work is to determine the best suitable parameters for producing the collars with exactly the required attribute. For this reason, a few precautionary measures and factors ought to be thought of. The essential benefit of this assessment work is to introduce a condition so that decreasing wastage material, lessening monetary loss and evade late shipment.

## 2 Experimental procedure

### 2.1 Materials and chemicals

20 Ne, 24 Ne, 28 Ne, 34 Ne and 40 Ne carded Techno Lot 2018A 100% cotton yarn were used to make Polo Pique for body fabrics and 1 × 1 rib fabric for collar. Reactive dyes, Tuscour HLF-18, Shuntex XPA, TF 208 C, IGLASTAB NSA, CLENATOL LAV, Caustic soda (NaOH), H2O2, CH3COOH, COENZYME-DL-75, OEM, IGLALEVEL BIP, TF 208 C, SHUNTEX XPA, salt was purchased from Bangladesh. All these chemicals were used as received, and no further purification was performed.

### 2.2 Knitting of Polo Pique fabric and flat knitted 1 x 1 Rib collar

Polo Pique fabric was knit on a circular knitting machine (Model: Mayer & CIE, Country of Origin: Germany), and the collar was knit on a v-bed flat knitting machine (Model: Shima Seiki, Country of Origin: Japan). V bed flat knitting machine with a gauge of 12 and a working width of 72 inches.

### 2.3 Pre-treatment

Different types of chemicals were used to do the scouring and bleaching of the fabric for the pretreatment and then the neutralization method. The pretreatment was carried out with a 1:8 material/liquor ratio in a dyeing machine (Sclavos, Greece). Different forms of auxiliaries, such as Tuscour hlf-18 (1.00 g/l), Shuntexxpa (0.80 g/l), Tf 208 c (1.00 g/l), Iglastabnsa (0.50 g/l), and Clenatollav (0.20 g/l), were applied to the dye bath with the fabric samples at 40 °C according to the amount of water used for continuing the method. The dyeing machine was then run for 5 min to increase the temperature to 98 °C. The dye bath was then treated with 2.00 g/L caustic soda and 3.50 g/L H2O2 (Hydrogen Peroxide). The system was then worked for another 40 min at 98 °C. After that, the temperature was lowered to 60 °C, and acetic acid 1.50 g/L, enzyme 0.50 percent, and peroxide killing agent 0.50 g/L were used to neutralize the peroxide.

### 2.4 Dyeing procedure

The body and 1 × 1 rib fabrics in this study are made entirely of 100% cotton yarn. Using the Sclavos dyeing system, the reactive dye was applied to both the body and the collar in a precise ratio. Cotton was dyed at higher temperatures, up to 60 °C, in the industrial dyeing process. At a pH of around 8.0, the M:L ratio was held at 1:8.

### 2.5 Finishing

**Figure 1:**
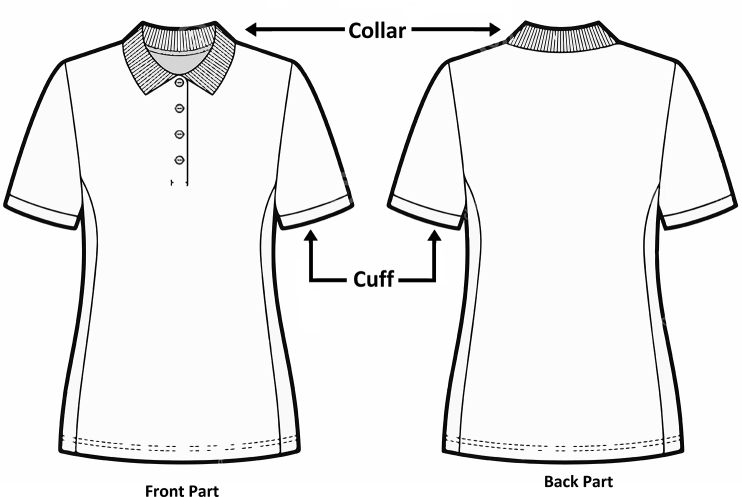
Schematic diagram of Polo Shirt.

**Figure 2:**
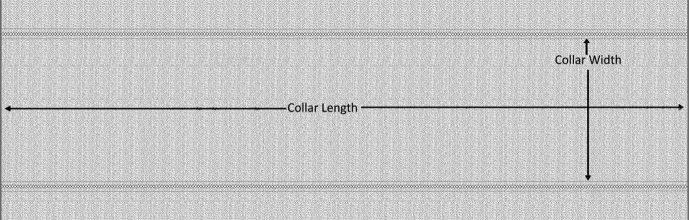
Cross sectional view of Collar (Body: Polo Pique and Collar: 1 × 1 Rib).

**Figure 3:**
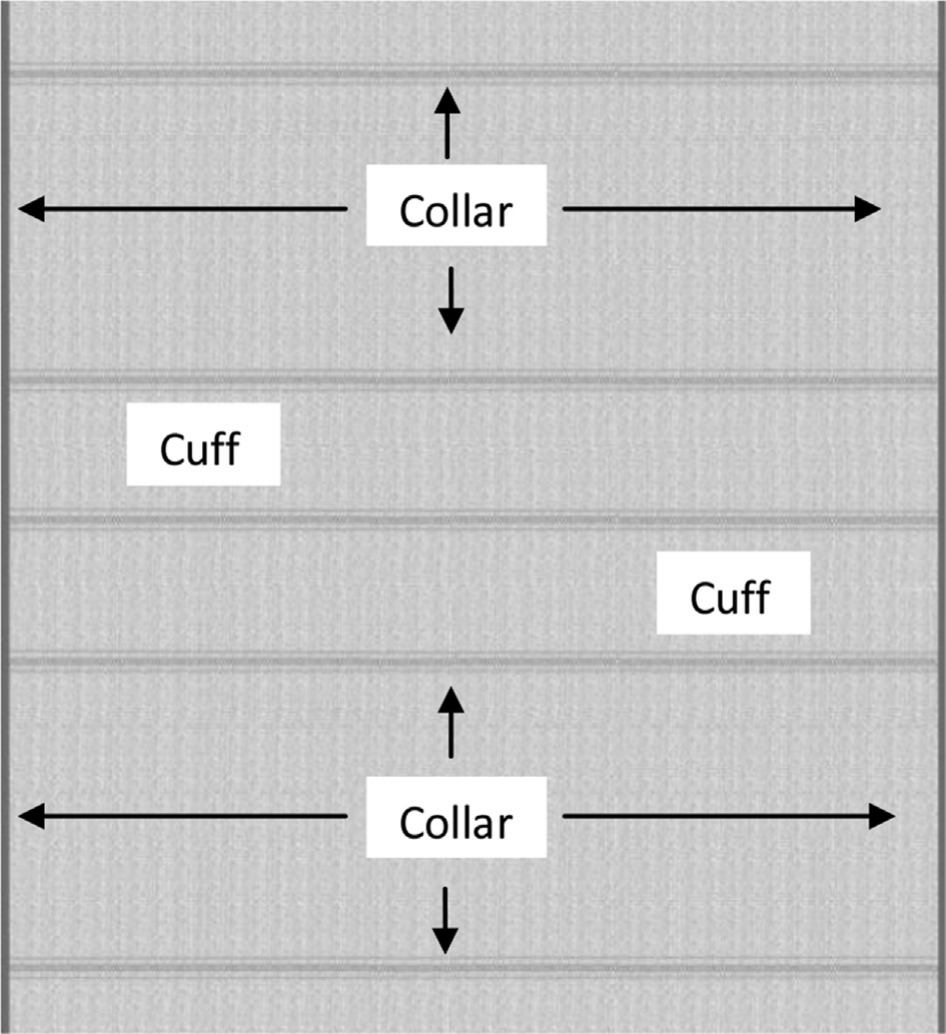
Grey Collar with Separation Thread.

**Figure 4:**
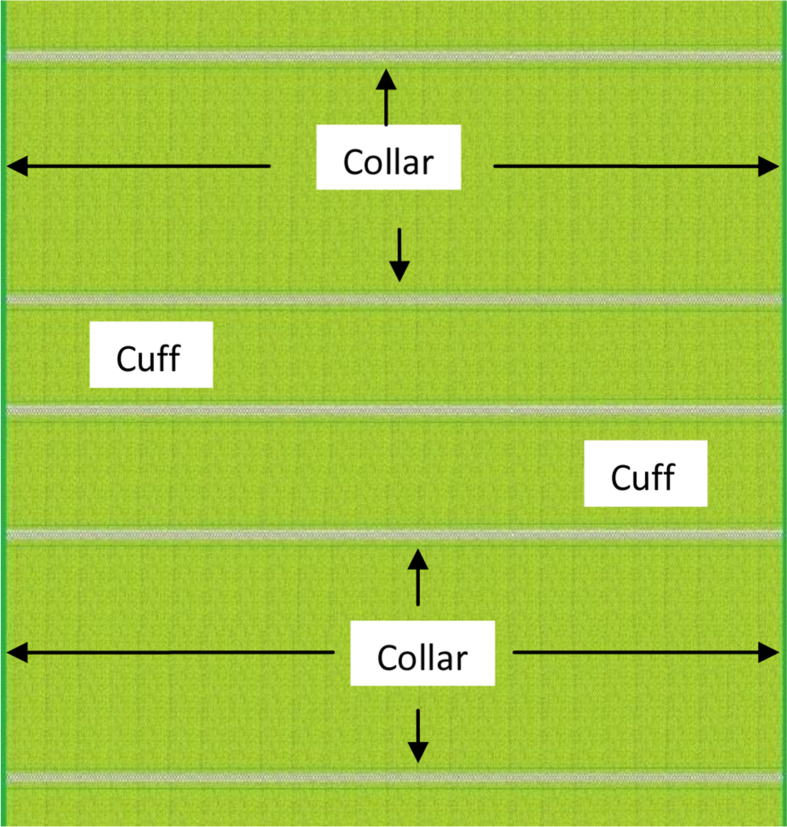
Dyed Collar with Separation thread.

Polo Pique (Figure 1) and flat knitted collars (Figure 2) and cuff (Figure 3) have different finishing methods. Slitting is achieved first for Polo Pique cloth, followed by drying in an open dryer, and then finishing with a compacting machine. The hydro extractor system, on the other side, extracts the water from the collar cloth, which is then dried in an open dryer machine. The study will only cover the collar finishing process since this research paper is focused on flat knitted collar cloth. Dysin from China, model no. KZ125, and a hydro extractor with a speed of 980 m/min were used in this research. On the other hand, a Santex (origin Switzerland, model no Santasphnink 3k/240gf1) dryer machine was used for drying, with a temperature of 130 °C and a speed of 50 m per minute and finally dyed collar and cuff are produced which is shown in Figure 4.

### 2.6 Testing

All of the experiments were carried out at a constant temperature of 21 degrees Celsius (70 degrees Fahrenheit) with a relative humidity of 62 percent. The samples were conditioned in a controlled environment for 2 h to determine their various parameters. The ASTM D3774 method was used to measure collar length and width. No external stress was applied to the samples as they were laid out. Following that, a ruler was used to measure the sample length and width. The width of the collar was measured perpendicular to the selvages, while the length was calculated parallel to the selvages. For both the length and width directions, measurements were taken in separate locations. The shrinkage of collar fabric was also tested in a controlled environment using the ISO 5077 test standard. After conditioning, the samples were measured according to ISO 139 and ISO 3759 procedures. Following that, the samples were washed and dried according to ISO 6330 procedures. After that, the sample fabrics were measured again, and the dimensional adjustments were determined using the ISO 3759 method. If the dimension was decreased (shrinkage), then it was denoted by a minus sign (-) or increased (extension) indicated by a plus sign (+). This formula was used to determine the value of spirality: ($spirality\;\%=\;\frac{x_{t}-x_{0}}{x_{0}}x100$), where xt = original dimension and x_0_ = dimension measured after treatment. GSM of the sample fabrics was measured using the D3773 test method. The sample fabrics were subjected to a conditioning procedure in order to assess their GSM. A steel tape was used to calculate the sample weight, and an electronic balance system was used to weigh it. The fabric sample was cut with a GSM cutter with a 100cm^2^ cutting area. The electronic balance was used to weigh the samples after they were cut. The GSM value of the samples was calculated by multiplying the value from the electronic balance by 100. To get the final GSM value, this process was repeated many times. The final GSM of the sample fabric was determined using the formula (Final GSM = Weight of Fabric from GSM cutter∗100). The sample fabric's stitch length was measured using the EN 14970:2006 testing method. 100 (hundred) wales were counted at first, and both of them were named. The yarns were then unrolled and the length of the unrolled yarn measured. After that, the stitch length was measured by the following formula: $\text{Stitch length}=\frac{\text{Measured length of }100\text{ wales in }\left(\text{mm}\right)}{\text{Total number of wales }\left(100\right)}$.

## 3 Result and discussion

### 3.1 Number of yarn ply selection

In this study, yarn counts of 20 Ne, 24 Ne, 28 Ne, 34 Ne, and 40 Ne were considered for sample creation. The number of yarn-plies used in collar knitting is critical because collar shrinkage, GSM, and knit capacity are all affected by the number of yarn-plies used. Since the GSM of the collar is higher than the GSM of the body cloth, knitting with a single ply cannot achieve the necessary collar GSM. To achieve the desired GSM, finer yarns usually need a greater number of plies. Table 1 shows that three plies are required to achieve the lowest shrinkage, target GSM, and improved knitting performance. In terms of shrinkage, GSM, and knitting performance, 4 ply for 24 Ne&28 Ne, 5 ply for 34 Ne, and 6 ply for 40 Ne yarn count are all suitable and specific number of needles are used for specific size which is shown in Table 2.

**Table 1:** Effect of number of ply on shrinkage, GSM and knit-ability.

Count	No. of Ply	Collar Shrinkage		Collar GSM	Collar Knit-ability
		Length wise shrinkage (%)	Width wise shrinkage (%)		
20 Ne	2	-3.5	-6.5	279	Loop Like Hole
	3	-2.5	-3	360	**No visual defect**
	4	-1.5	-2.5	425	hole
24 Ne	3	-3.7	-6.4	343	Loop Like Hole
	4	-2.4	-2.9	399	**No visual defect**
	5	-1.4	-2.4	432	Pin hole
28 Ne	3	-3.8	-6.4	325	Loop Like Hole
	4	-2.5	-2.9	380	**No visual defect**
	5	-1.2	-2.4	412	hole
34 Ne	4	-3.4	-6.4	360	Loop Like Hole
	5	-5.3	-2.9	385	**No visual defect**
	6	-1.4	-2.4	500	Pin hole
40 Ne	5	-4.4	-7.1	378	Loop Like Hole
	6	-2.5	-4	488	**No visual defect**
	7	-1.5	-2.4	508	Pin hole

**Table 2:** Number of needles used for different collar size.

Collar size (cm)	Total needle used
36	252
37	257
38	262
39	267
40	272
41	277
42	282
43	287
44	292
45	297
46	302
47	307
48	312
49	317
50	322

### 3.2 Stitch length selection

**Figure 5:**
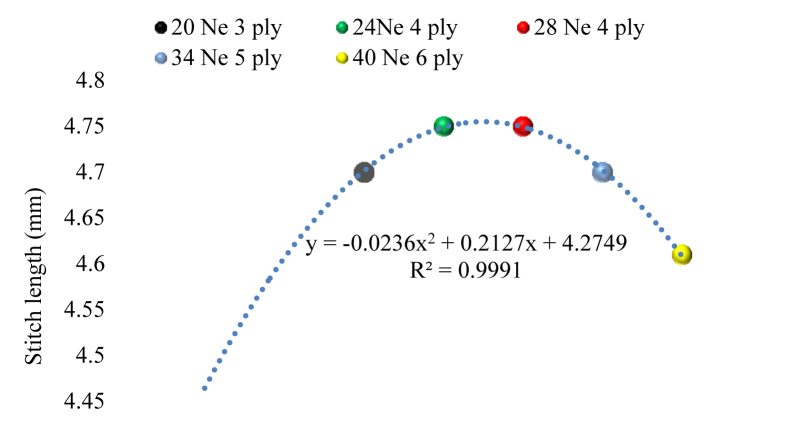
Suitable stitch length perspective to different yarn count and ply.

Figure 5 illustrates the required stich length for different yarn count and yarn ply combination.

The graph shows that as the yarn ply increased, the necessary stitch length increased (4.7 mm, 4.75 mm, and 4.76 mm, respectively) for 20, 24, and 28 Ne count. After that, there was a reversal of the pattern. As the yarn fineness and the amount of ply increased, the required stitch length decreased until it reached 4.6 mm. An oval shape line diagram was derived having the equation of

(i) y = -0.0236x^2^ + 0.2127x + 4.2749 and R^2^ = 0.9991

### 3.3 Needle selection

The smallest collar size is 36 cm, and the upper collar size is 50 cm, which is the most common size in the industry. Since the collar can stretch due to relaxation after separation thread removal and applied pressure during collar attachment in the sewing machine, the finished collar size should be 0.4 cm–1.2 cm smaller than the necessary size. A formula has been introduced to determine the total number of needles to achieve exact collar measurement, keeping the finished collar size 0.4 cm–1.2 cm smaller than the appropriate collar size.

**Formula:** Based on the experimental results we have recorded the number of total needles used against different collar size. The recorded data has been enlisted in Table 2.

A graph has been illustrated by plotting the collar size in X axis and the required needle in the Y axis. The graph represented a straight-line diagram with a linier equation:

(ii) N = 5X+72

where, N = Total needle required.

X = required collar size (cm).

**Figure 6:**
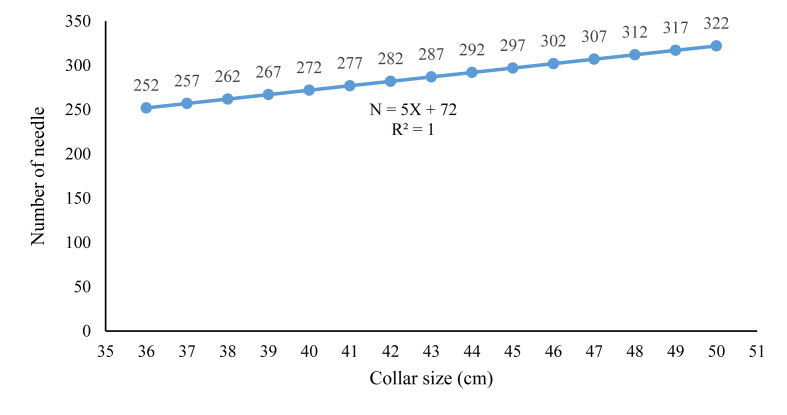
Total needle used with respect to different collar size.

Figure 6 represents the required number of needle against different collar size.

### 3.4 Shrinkage value of the developed sample

**Figure 7:**
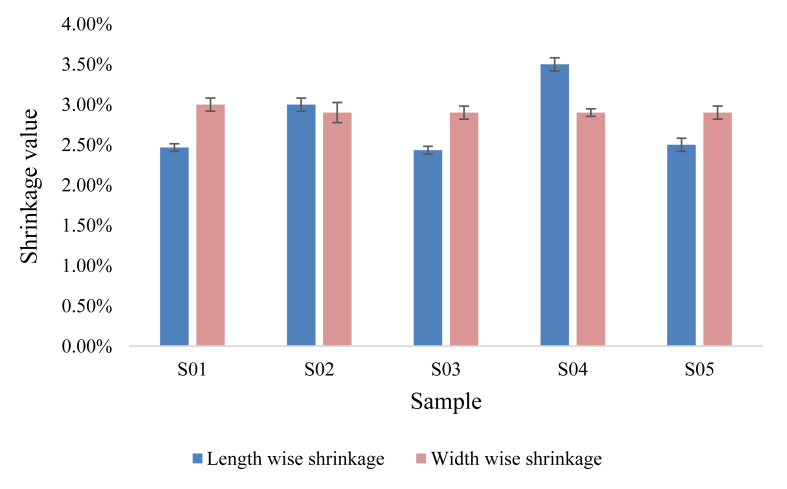
Length and width wise shrinkage value of the developed samples.

The shrinkage values of the samples are shown in Figure 7 in both length and width directions. Every criteria of the individual sample was registered three times. The mean value was shown in the bar graph, and the standard deviation was shown as the error bar.

The shrinkage was irregular in both length and width. The width-wise shrinkage was higher than the length-wise shrinkage in the S01, S03, and S05 tests, and the length-wise shrinkage was higher than the width-wise shrinkage in the rest of the cases. Many of the samples, however, had shrinkage values ranging from 2.5 to 3.5 percent.

### 3.5 GSM value of the developed samples

**Figure 8:**
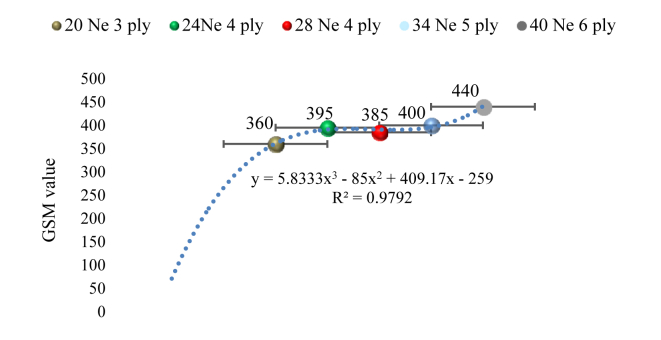
GSM value of the developed simples.

Figure 8 illustrates the change of GSM concerning different yarn count and number of ply. The standard deviation was represented as the error bar. It is clear from the graph that the collar GSM is proportional to the number of ply and it can be expressed by the equation

(iii) y = 5.8333x^3^ - 85x^2^ + 409.17x – 259

The GSM value propositionally increased with the increase in the number of ply, starting at 350 for a 20 Ne 3 ply sample. The increased yarn thickness caused by the multiple ply resulted in a higher GSM. The GSM value increased to 395 for 20 Ne 3 ply. Since the fineness of the yarn was raised while the yarn ply remained unchanged, it was further reduced to 385 for 28 Ne 4 ply. Following that, the GSM value continued to rise, reaching a peak of 440 for 40 Ne 6 ply.

### 3.6 Required size and finished size of the samples

**Figure 9:**
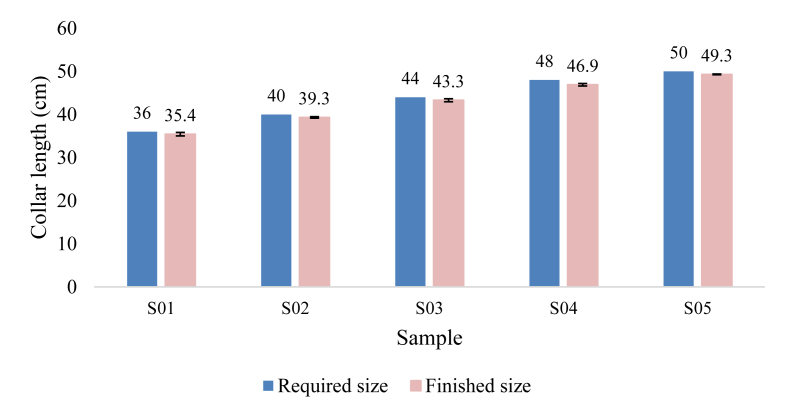
Required and finished collar size of the samples.

Figure 9 compares the required size and the finished size of the collar. Data was recorded three times for each criterion of the individual sample. The bar graph was demonstrated with the mean value, and the standard deviation was presented as the error bar.

For S01, S02, S03 & S05 samples, the difference was between 0.6 and 0.7 cm. Only for the S04 sample, the difference was 1.1 cm. It was evident that the finished size is less than the required size, which is commonly expected as the collar size increases when it gets relaxed.

## 4 Conclusion

{20 Ne, 3 ply, 4.7 mm}, {24 Ne, 4 ply, 4.75 mm}, {28 Ne, 4 ply, 4.76 mm}, {34 Ne, 5 ply, 4.68 mm}, {40 ne, 6 ply, 4.6 mm} are the best combinations in terms of yarn count, number of yarn ply and stitch length for collar manufacturing in v-bed knitting machine. The number of needle used in knitting can be derived according to the equation of 5x+72 where “x” is the required collar size in centimeter. This research work removes the complexity for selecting stitch length, the number of plies, grey size, finished size, and needle of a final collar. It can be a proper guideline for the collar manufacturers to solve their practical problems during the manufacturing process.

## Declarations

### Author contribution statement

Md. Shakhawat Hossain: Conceived and designed the experiments; Performed the experiments.

MD. Momtaz Islam: Analyzed and interpreted the data; Wrote the paper.

Naimul Hasan: Analyzed and interpreted the data; Contributed reagents, materials, analysis tools or data.

### Funding statement

This research did not receive any specific grant from funding agencies in the public, commercial, or not-for-profit sectors.

### Data availability statement

Data will be made available on request.

### Declaration of interests statement

The authors declare no conflict of interest.

### Additional information

No additional information is available for this paper.
